# Preferred Social Media Platform for Rehabilitation Education Following Anterior Cruciate Ligament Reconstruction

**DOI:** 10.7759/cureus.107851

**Published:** 2026-04-27

**Authors:** Abdullah H AlMuhaya, Mai Aldera, Dalia Alimam

**Affiliations:** 1 Department of Sport Rehabilitation, Joint Clinics, Medical Rehabilitation Center, Riyadh, SAU; 2 Department of Health Rehabilitation Sciences, College of Applied Medical Sciences, King Saud University, Riyadh, SAU

**Keywords:** anterior cruciate ligament reconstruction, digital health, patient education, patient preference, rehabilitation, saudi arabia, social media, video-based education

## Abstract

Introduction: Designing effective digital rehabilitation educational content requires alignment between platform functionality and the patient’s digital behavior. However, there is limited evidence regarding the platforms selected for structured rehabilitation education following anterior cruciate ligament reconstruction (ACLR). Therefore, this study aimed to identify the social media platforms (SMPs) preferred by patients who underwent ACLR to guide the design of digital educational rehabilitation strategies in Saudi Arabia.

Materials and methods: From November 2024 to January 2025, 186 participants (83.3% men) who met the following inclusion criteria were included: aged 18 years or older, underwent ACLR surgery, used more than one SMP, were currently undergoing or had completed ACLR rehabilitation, and responded to an online survey shared by their physiotherapy and sports rehabilitation clinics. The participants’ responses were analyzed using descriptive statistics, Cochran’s Q test, and chi-square tests.

Results: Preferences for SMP use differed significantly (p < 0.001), with most participants preferring Snapchat (61.3%). Men demonstrated higher use of Snapchat and TikTok compared with women (p < 0.05); however, no significant differences were observed across age groups and frequency of platform use. Video-based content was the most frequently preferred format (86.6%, 161/186), with chi-square analysis showing a significant association with preference for Snapchat (p < 0.001).

Conclusion: These findings indicate that patients undergoing rehabilitation following ACLR prefer video-based educational content on Snapchat. These findings suggest that future educational strategies should explore the use of Snapchat to deliver brief, engaging, and culturally tailored rehabilitation materials. Future research should evaluate whether platform-aligned education can improve engagement and rehabilitation outcomes.

## Introduction

Anterior cruciate ligament (ACL) injuries are among the most frequent and serious knee injuries sustained during sports participation [1,2]. ACL injuries mainly affect young, active individuals, especially those who participate in pivoting and high-impact sports [1,2]. Therefore, ACL reconstruction (ACLR) has become one of the most commonly performed orthopedic procedures worldwide to restore knee stability and facilitate return to sport (RTS) [1,2].

Patient education is a key factor influencing successful recovery and adherence to rehabilitation programs in patients with ACL injuries [3]. Traditional approaches rely on face-to-face consultations and printed materials, which are often limited by consultation-time constraints and lack of ongoing reinforcement after clinic discharge [3]. Digital health approaches, such as online videos, provide visual and accessible learning experiences [4,5]. Platforms such as Snapchat and TikTok operate as micro-video tools that deliver brief, visual, accessible, and demonstration-based educational content suitable for rehabilitation needs, allowing the convenient sharing of health information and supporting patient engagement [6,7].

Saudi Arabia has undergone rapid digital adoption, providing alternative channels for health education. National statistics report an Internet penetration of 99% and that approximately 80% of users engage with at least one social media platform (SMP) [8,9]. Among the most widely used SMPs in the Kingdom of Saudi Arabia (KSA) are YouTube, TikTok, Snapchat, Instagram, and Facebook [9]. About one-third of online activity involves searching for health information, such as exercise programs, rehabilitation, injury prevention, and pain management [10]. Additionally, more than 90% of Saudis believe that SMPs enhance health awareness [10].

Although SMPs provide accessible channels for health information, the reliability of content varies considerably. Studies assessing general health-related content on social media report that a substantial proportion of posts lack professional validation or scientific accuracy, which may contribute to confusion, poor treatment adherence, and increased anxiety among patients [11-13]. Evidence specific to musculoskeletal or ACL rehabilitation content remains limited, highlighting the need to examine the reliability of educational content delivered to these populations. Conversely, when used effectively, SMPs can deliver interactive and accessible education, support communication, and provide motivation during rehabilitation [13-16]. The increasing role of SMPs in KSA's healthcare landscape necessitates understanding which platforms are most suitable for structured patient education, particularly for conditions requiring long-term rehabilitation, such as ACLR.

In current rehabilitation settings, patient education is typically provided face-to-face during clinic visits, with limited continuity when patients return home. Although many clinicians supplement care using digital messages or online videos, there is no standardized or evidence-based approach for delivering structured educational content through SMPs [15,17]. This gap is particularly relevant for ACLR, as patients often seek information independently online to manage pain, restore function, and prepare for RTS.

Despite the widespread use of SMPs for health information [10,14], there is limited evidence regarding the SMPs that patients who underwent ACLR prefer for structured, credible, and interactive educational content. Therefore, understanding SMP preferences is crucial for developing targeted and patient-centered educational interventions that align with the patients' digital habits [15,17,18].

Identifying SMP preferences is a logical first step because educational delivery methods aligned with patient digital behavior are hypothesized to support learning, motivation, and adherence. Educational interventions have been associated with improved psychological readiness, reduced kinesiophobia, and greater adherence to home exercise programs, which may in turn influence successful rehabilitation and RTS [1,2,15,16]; however, whether platform-aligned digital education can produce these effects in ACLR populations remains to be tested. In Saudi rehabilitation settings, providing structured educational material during ACL rehabilitation has been shown to be feasible and has been associated with improved confidence and psychological readiness for RTS [19]. Characterizing patients' preferred SMPs is therefore a necessary precursor to designing platform-aligned educational interventions that can subsequently be evaluated in controlled studies.

The primary aim of this study was to identify the SMPs preferred by patients who underwent ACLR for receiving rehabilitation-related educational content in Saudi Arabia. Secondary aims were to examine (i) associations between preferred platforms and participant sex and age and (ii) the relationship between platform preference and preferred educational content format. We hypothesized that platform preferences would differ significantly across the available SMPs and that preferences would vary by sex and by preferred content format, while remaining comparable across age groups, given the largely young adult composition of the ACLR population.

## Materials and methods

This study employed a cross-sectional design and was reported in accordance with the Strengthening the Reporting of Observational Studies in Epidemiology (STROBE) statement for cross-sectional studies. Data were collected from patients who underwent ACLR using a self-reported online survey. A completed STROBE checklist is provided as Supplementary Material 1.

Inclusion and exclusion criteria

Individuals who underwent or were currently undergoing ACLR rehabilitation, were aged 18 years or older, understood Arabic, were willing to participate and provide informed consent, and used more than one SMP were included. Participants were excluded if they used only one SMP or had undergone surgical procedures other than ACLR.

Data collection

Convenience sampling was used. Electronic invitations were distributed in collaboration with ten private, cash-based physiotherapy and sports rehabilitation outpatient clinics specialized in musculoskeletal and sports injury care, located across four major cities in Saudi Arabia: Riyadh, Jeddah, Dammam, and Abha. Invitations were shared with potential participants through the clinics' patient communication channels, including email lists, messaging platforms, social media accounts, and in-clinic advertisements, between November 2024 and January 2025. The survey was hosted on Google Forms (Google LLC, Mountain View, CA, USA), and the link was publicly accessible to eligible patients until the a priori sample size target was reached, at which point form responses were closed. Because participation occurred via an open link without individual invitation, a conventional response rate could not be calculated; this is acknowledged as a limitation. Data were stored in the principal investigator's Google Drive and were accessible only to the research team following approval from the Institutional Review Board of the College of Medicine, King Saud University (KSU 24/1757/IRB). No personal identifiers were collected. Duplicate submissions were prevented by restricting one response per Google account and screening for similarities across responses.

Participant instructions

The participants received a brief overview of the study, screening questions, notification of voluntary participation, informed consent, and confidentiality assurance. Instructions for navigating and completing the survey were provided on the form.

Ethical approval

Ethical approval was obtained from the Institutional Review Board of the College of Medicine, King Saud University (reference number: 24/1757/IRB).

Online survey

The survey was developed by adapting items from two prior Saudi studies examining social media use for health-related information [18,20]. Specifically, items addressing platform familiarity and frequency of use were drawn from Marar et al. [20], and items addressing professional use of social media for health information were drawn from Alsobayel [18]. These items were modified to align with the present study's focus on ACLR rehabilitation education, with contextual adjustments to terminology (e.g., replacing general "health information" references with "rehabilitation education") and the addition of items specific to content format preference (videos, infographics, articles, live sessions) and trust in platform-delivered educational content. Before being used, the research team checked the adapted instrument for face validity against the study goals.

It comprised four sections. The first section collected sociodemographic data, including age group, sex, and physical activity before and after injury. Physical activity was reported using four predefined categories (inactive, light, moderate, or intense). The second section assessed patterns of SMP use. Participants identified the SMPs they used, the average number of hours spent daily using SMPs, and the weekly time spent accessing health-related information. They also identified the platforms on which they preferred to receive health-related content. The questions in this section were multiple-choice questions, with some allowing for multiple selections.

The third section evaluated preferences regarding the educational content of the SMPs. Participants selected their preferred content formats (e.g., videos, infographics, articles, or live sessions) and the main reasons for their platform preferences, such as ease of use, content variety, popularity, visual quality, and professional communication. Categorical responses and multiple selection options were used for each item. The final section examined the perceptions of and trust in health-related information on SMPs. Participants were asked whether they trusted the accuracy of the information on SMPs and the reasons for their level of trust. The participants were also asked about their beliefs regarding the educational value of SMPs during ACL rehabilitation. These questions used yes/no/maybe or categorical responses. All questions were closed-ended with predefined answer options, and certain items allowed for the selection of more than one response (Supplemental Material 1).

Statistical analysis

Data were analyzed using IBM SPSS Statistics for Windows, Version 31 (Released 2025; IBM Corp., Armonk, New York). Preference variables were coded as 1 (selected) or 0 (not selected) for each platform. All variables were summarized using descriptive statistics. Because participants could select multiple SMPs, Cochran's Q test was used to evaluate differences in platform preferences; when significant, post-hoc McNemar tests with Holm correction for multiple comparisons were applied. Exploratory analyses assessed the associations between preferred platforms and participant age, sex, and preference for video-based content using Pearson chi-square tests. Effect sizes for significant chi-square associations were reported as Cramér's V, interpreted according to Cohen's conventions (small ≈ 0.10, medium ≈ 0.30, large ≈ 0.50) [21]. Given the descriptive aim of characterizing platform preferences and the limited statistical reliability of multivariable modeling in a subgroup of 31 female participants, multivariable logistic regression was not performed; potential confounding between sex and age is acknowledged as a limitation. All tests were two-tailed, with significance set at p < 0.05.

Sample size calculation

Sample size was calculated using G*Power software version 3.1 (Heinrich Heine University Düsseldorf, Düsseldorf, Germany) for a χ² goodness-of-fit test to detect differences across SMP preferences. In the absence of prior studies reporting effect sizes for SMP preference distributions in ACLR populations, Cohen's conventional medium effect size of w = 0.30 was adopted [21], with α = 0.05 and power = 0.80. Considering seven SMP categories (YouTube, TikTok, Snapchat, WhatsApp, Twitter/X, Facebook, and Instagram), the degrees of freedom were set at 6 (df = k − 1), yielding a minimum required sample of 152. Allowing for a 20% non-response rate, the target was increased to 182. A total of 186 participants were recruited, meeting the adjusted sample size requirement.

## Results

Demographics and sample characteristics

In total, 186 participants completed the survey and were included in the final analysis (Table 1). The sample was predominantly male (83.3%, n = 155), with only 31 female participants (16.7%), an imbalance that should be considered when interpreting sex-based analyses. The largest age group was 26-35 years (47.8%, n = 89), followed by 18-25 years (26.3%, n = 49) and 36-45 years (20.4%, n = 38). Only 10 participants (5.4%) were aged 46 years or older. Physical activity data are reported in Table 1 to describe the population and to illustrate how participants' activity levels changed following ACL injury, rather than as variables entering inferential analysis. Before the ACL injury, 37.1% (n = 69) of participants reported intense physical activity, and 9.1% (n = 17) were sedentary. After the injury, sedentary behavior increased to 18.8% (n = 35) and intense activity declined to 10.8% (n = 20), reflecting the expected impact of ACL injury on physical activity levels.

**Table 1:** Demographic Characteristics and Physical Activity of Participants (N = 186)

Variable	Category	Frequency (n)	Percentage (%)
Sex	Male	155	83.3
	Female	31	16.7
Age group	18–25	49	26.3
	26–35	89	47.8
	36–45	38	20.4
	46–55	8	4.3
	56 and more	2	1.1
Physical activity before injury	Sedentary	17	9.1
	Light	49	26.3
	Moderate	51	27.4
	Intense	69	37.1
Physical activity after injury	Sedentary	35	18.8
	Light	73	39.2
	Moderate	58	31.2
	Intense	20	10.8

SMP usage

Most participants spent 2-4 hours daily using SMPs (46.2%, n = 86), while only one participant reported ≤1 hour of usage. For health-related information seeking on SMPs, 40.9% (n = 76) reported ≤1 hour per week, while 7.0% (n = 13) did not use SMPs for this purpose.

Participants most frequently cited ease of use as a reason for platform preference (81.7%, 152/186), followed by content variety (41.4%, 77/186), platform popularity (36.6%, 68/186), and user engagement (22.0%, 41/186). Professional communication was cited less frequently (11.3%, 21/186), and 14.0% (26/186) did not prefer using SMPs for health information. Because this was a multi-select item, percentages exceed 100% (see Table 2 note).

**Table 2:** Patterns of Social Media Platform (SMP) Usage, Preferences, Trust, and Perceived Educational Value (N = 186) Note: ᵃ For multi-select items, percentages represent the proportion of participants (out of N = 186) who selected each option and therefore do not sum to 100%. For single-response variables, percentages sum to 100% within each variable, allowing for rounding.

Variable	Category	Frequency (n)	Percentage (%)
Hours spent daily using SMPs	2–4 hours	86	46.2
	5–7 hours	77	41.4
	8 hours or more	22	11.8
	One hour or less	1	0.5
Hours spent weekly searching/reading for health-related information using SMPs	2–4 hours	72	38.7
	5–7 hours	20	10.8
	8 hours or more	5	2.8
	One hour or less	76	40.9
	I do not use SMP for health information	13	7.0
Main reasons for platform preference (multi-select)ᵃ	Ease of use	152	81.7
	Content variety	77	41.4
	Popularity	68	36.6
	User engagement	41	22.0
	I do not prefer to use SMP for health information	26	14.0
	Professional communication	21	11.3
Preferred content type (multi-select)ᵃ	Videos	161	86.6
	I do not seek to share/receive health information through SMP	25	13.4
	Infographics	14	7.5
	Articles	22	11.8
	Live sessions (webinars)	15	8.1
Trust in SMP information	Yes	49	26.3
	No	16	8.6
	Maybe (Uncertain)	106	57.0
	Trust depends on the source's credibility	15	8.1
Reason for trust	A well-known person increases my trust in the information	147	79.0
	I generally trust.	35	18.8
	I do not trust medical information on social media platforms	4	2.2
Belief in educational use of SMPs	Yes	159	85.5
	No	27	14.5

Video-based content was the most frequently preferred educational format (86.6%, 161/186), while infographics were the least preferred (7.5%, 14/186). Articles (11.8%, 22/186) and live sessions (8.1%, 15/186) were preferred by smaller subsets. A minority of participants (13.4%, 25/186) indicated they did not use SMPs to receive or share health information.

Trust in information from SMPs varied: 26.3% (n = 49) reported that they trusted the information, 57.0% (n = 106) were uncertain, 8.6% (n = 16) did not trust it, and 8.1% (n = 15) indicated that their trust depended on the perceived credibility of the source. Among the factors influencing trust, the presence of a well-known or credible source was the most frequently cited (79.0%, n = 147). Overall, 85.5% (n = 159) believed that SMPs could be effectively used for educational purposes, whereas 14.5% (n = 27) did not (Table 2).

Exploratory analyses examining associations between trust variables and platform preference showed that Snapchat preference was significantly associated with higher overall trust in SMP health information (χ²(3) = 7.97, p = 0.047, Cramér's V = 0.21) and with citing "a well-known person" as the main driver of trust (χ²(2) = 8.77, p = 0.012, Cramér's V = 0.22). No significant trust-related associations were observed for the other platforms.

Familiarity, daily use, and preferred SMPs

Participants showed high familiarity with most SMPs (Table 3). Snapchat was the most commonly used platform (96.2%, n = 179), whereas Instagram (40.9%, n = 76) and Facebook (14.5%, n = 27) were the least used. Daily usage followed a similar pattern, with Snapchat (95.7%, n = 178) being the most used and Facebook (14.5%, n = 27) the least. Regarding the platforms preferred for receiving health-related information, Snapchat was preferred by 61.3% (n = 114) of participants, whereas Twitter/X (7.5%, n = 14) and WhatsApp (5.4%, n = 10) were the least preferred; Facebook was not selected at all.

**Table 3:** Familiarity, Daily Use, and Preferred Social Media Platforms (SMPs) Among Participants (N = 186) Note: Totals exceed 100% because participants could report familiarity, daily use, and preference for more than one platform.

Platform	Familiarity N (%)	Daily Use N (%)	Preference N (%)
YouTube	153 (82.3)	149 (80.1)	69 (37.1)
TikTok	161 (86.6)	156 (83.9)	48 (25.8)
Snapchat	179 (96.2)	178 (95.7)	114 (61.3)
WhatsApp	164 (88.2)	159 (85.5)	10 (5.4)
Twitter/X	156 (83.9)	151 (81.2)	14 (7.5)
Facebook	27 (14.5)	27 (14.5)	0 (0.0)
Instagram	76 (40.9)	74 (39.8)	38 (20.4)

Comparison of familiarity, daily use, and preference data revealed a notable discrepancy. WhatsApp showed high familiarity (88.2%) and daily use (85.5%), yet was preferred for health information by only 5.4% of participants. A similar, smaller gap was observed for YouTube (familiarity 82.3%; preference 37.1%) and TikTok (familiarity 86.6%; preference 25.8%). In contrast, Snapchat's preference (61.3%) closely followed its familiarity (96.2%) and daily use (95.7%). This pattern suggests that general familiarity with a platform did not uniformly translate into preference for health-related educational content, and that additional factors, such as content format fit or perceived credibility, may drive platform selection for rehabilitation education specifically.

Preferred SMP (primary outcome)

SMP preferences varied by age and sex (Table 4). Snapchat was the leading platform across all age groups, being the most preferred among younger participants (63.3% in 18-25 years and 62.9% in 26-35 years) and remaining dominant in middle-aged groups (55.3% in 36-45 years and 62.5% in 46-55 years). Due to the small size of the ≥56 years subgroup (n = 2), descriptive preferences are reported for completeness but were not interpreted inferentially. Snapchat was the most frequently selected platform overall (61.3%). Men favored Snapchat (65.8%) and TikTok (29.0%), whereas women reported lower Snapchat preference (38.7%) but a relatively higher preference for Instagram (22.6%). Facebook received zero selections and was therefore excluded from inferential analysis (Table 4).

**Table 4:** Preferred Social Media Platforms (SMPs) by Age Group and Sex (N = 186) Note: ᵃ The ≥56 years subgroup is reported descriptively only; due to insufficient size (n = 2), it was excluded from inferential testing. Percentages within each age or sex subgroup do not sum to 100% because participants could select more than one preferred platform.

Age/Sex	YouTube n (%)	TikTok n (%)	Snapchat n (%)	WhatsApp n (%)	Twitter/X n (%)	Instagram n (%)
18–25 yrs (n=49)	15 (30.6)	13 (26.5)	31 (63.3)	3 (6.1)	3 (6.1)	8 (16.3)
26–35 yrs (n=89)	37 (41.6)	22 (24.7)	56 (62.9)	2 (2.2)	4 (4.5)	25 (28.1)
36–45 yrs (n=38)	13 (34.2)	10 (26.3)	21 (55.3)	3 (7.9)	7 (18.4)	5 (13.2)
46–55 yrs (n=8)	2 (25.0)	2 (25.0)	5 (62.5)	2 (25.0)	0 (0.0)	0 (0.0)
≥56 yrs (n=2)ᵃ	2 (100.0)	1 (50.0)	1 (50.0)	0 (0.0)	0 (0.0)	0 (0.0)
Male (n=155)	61 (39.4)	45 (29.0)	102 (65.8)	7 (4.5)	11 (7.1)	31 (20.0)
Female (n=31)	8 (25.8)	3 (9.7)	12 (38.7)	3 (9.7)	0 (0.0)	7 (22.6)
Total (N=186)	69 (37.1)	48 (25.8)	114 (61.3)	10 (5.4)	14 (7.5)	38 (20.4)

Cochran's Q test (Table 5) showed that SMP preferences differed significantly in the total sample (Q = 201.24, df = 5, p < 0.001), indicating clear and distinct preference patterns. Significant differences were also observed in men, women, and across the three main age categories (18-25, 26-35, and 36-45 years); the 46-55 group showed a weaker but still significant result, while the ≥56 subgroup (n = 2) was excluded from inferential testing due to insufficient size.

**Table 5:** Cochran’s Q Test Results for Overall and Demographic Subgroups *p < 0.05. Note: Cochran's Q test assesses whether the proportions of participants selecting each platform differ significantly within each subgroup. Degrees of freedom vary because only platforms selected in a subgroup were included in that subgroup's analysis. The ≥56 years subgroup (n = 2) was excluded from inferential testing due to insufficient size; descriptive data are retained in Tables 1 and 4.

Subgroup	Q statistic	df	*p*-value
Total sample (N = 186)	201.24	5	<0.001*
Age 18–25 y (n = 49)	75.42	5	<0.001*
Age 26–35 y (n = 89)	83.15	5	<0.001*
Age 36–45 y (n = 38)	42.87	5	<0.001*
Age 46–55 y (n = 8)	10.12	4	0.038*
Male (n = 155)	176.30	5	<0.001*
Female (n = 31)	29.18	4	<0.001*

Post-hoc McNemar tests with Holm corrections (Table 6) indicated that Snapchat was selected significantly more often than every other platform. YouTube was selected significantly more often than WhatsApp, Twitter/X, or Instagram. TikTok was preferred over WhatsApp and Twitter/X. The comparison between YouTube and TikTok did not reach statistical significance after Holm correction (adjusted p = 0.072), suggesting that these two platforms may play overlapping roles as secondary preferences in rehabilitation education. No significant differences were detected between TikTok and Instagram or between WhatsApp and Twitter/X.

**Table 6:** Pairwise McNemar Tests for Preferred SMPs With Holm-Adjusted P-values (N = 186) * Statistically significant after Holm correction (p < 0.05). Note: McNemar tests compare paired proportions of platform selections. Raw p-values were adjusted for multiple testing using both Bonferroni and Holm procedures; significance was determined using the Holm-adjusted p < 0.05. “Y” indicates significance after Holm adjustment. “–” indicates that the comparison could not be estimated because of zero discordant pairs.

#	Platform A	Platform B	McNemar χ² (df = 1)	Raw *p*-value	Bonferroni *p*-value	Holm-adjusted *p*-value	Significant after Holm (Y/N)
1	Snapchat	YouTube	27.27	<0.001*	0.015*	0.014*	Y
2	Snapchat	TikTok	58.68	<0.001*	0.015*	0.013*	Y
3	Snapchat	WhatsApp	91.46	<0.001*	0.015*	0.012*	Y
4	Snapchat	Twitter/X	80.34	<0.001*	0.015*	0.011*	Y
5	Snapchat	Instagram	48.49	<0.001*	0.015*	0.010*	Y
7	YouTube	WhatsApp	42.58	<0.001*	0.015*	0.009*	Y
8	YouTube	Twitter/X	41.07	<0.001*	0.015*	0.008*	Y
9	YouTube	Instagram	12.33	<0.001*	0.015*	0.007*	Y
10	TikTok	WhatsApp	24.45	<0.001*	0.015*	0.006*	Y
11	TikTok	Twitter/X	17.57	<0.001*	0.015*	0.005*	Y
14	WhatsApp	Instagram	15.85	<0.001*	0.015*	0.004*	Y
6	YouTube	TikTok	5.06	0.024	0.36	0.072	N
12	TikTok	Instagram	0.96	0.326	1.00	0.652	N
13	WhatsApp	Twitter/X	–	0.481	1.00	0.481	N
15	Twitter/X	Instagram	–	–	–	–	N/not estimable

Association analysis

Chi-square tests (Table 7) showed significant sex-based differences for TikTok (χ²(1) = 5.05, p = 0.025, Cramér's V = 0.17) and Snapchat (χ²(1) = 8.00, p = 0.005, Cramér's V = 0.21), with men being more likely to prefer both platforms. These effect sizes are small to small-to-medium, and results should be interpreted with caution given the limited size of the female subgroup (n = 31). No significant sex-based differences were found for YouTube, WhatsApp, Twitter/X, or Instagram.

**Table 7:** Association Between Sex and Preferred Social Media Platforms (SMPs) (N = 186) * p < 0.05. Note: Cramér's V values were interpreted per Cohen's conventions (small ≈ 0.10, medium ≈ 0.30, large ≈ 0.50). Findings involving the female subgroup (n = 31) should be interpreted as preliminary due to small cell counts.

Preferred SMP	Male n (%)	Female n (%)	Total n (%)	χ² (df)	p-value	Cramér's V
YouTube	61 (39.4)	8 (25.8)	69 (37.1)	2.03 (1)	0.154	0.10
TikTok	45 (29.0)	3 (9.7)	48 (25.8)	5.05 (1)	0.025*	0.17
Snapchat	102 (65.8)	12 (38.7)	114 (61.3)	8.00 (1)	0.005*	0.21
WhatsApp	7 (4.5)	3 (9.7)	10 (5.4)	1.35 (1)	0.245	0.09
Twitter/X	14 (9.0)	0 (0.0)	14 (7.5)	3.03 (1)	0.082	0.13
Instagram	29 (18.7)	9 (29.0)	38 (20.4)	1.69 (1)	0.193	0.10

When the ≥56 years subgroup (n = 2) was excluded from inferential testing, no robust age-based associations with platform preference were observed (Table 8). WhatsApp and Twitter/X showed marginal p-values (0.042 and 0.039, respectively); however, these results should be interpreted with caution because chi-square assumptions were violated (>25% of cells had expected counts <5 and at least one cell had an expected count <1 for both comparisons), reflecting the low overall preference rates for these platforms.

**Table 8:** Association Between Age Group and Preferred Social Media Platforms (SMPs) (N = 184) * p < 0.05. ᵃ Chi-square assumptions were violated for WhatsApp and Twitter/X (≥38% of cells with expected counts <5, and at least one cell with expected count <1); these p-values should therefore be interpreted with caution as they likely reflect low overall preference rates for these platforms rather than meaningful age-based associations. Note: The ≥56 years subgroup (n = 2) was excluded from inferential testing due to insufficient size; descriptive data are retained in Tables 1 and 4. Percentages within each age subgroup do not sum to 100% because participants could select more than one preferred platform.

Preferred SMP	18–25 yrs (n=49)	26–35 yrs (n=89)	36–45 yrs (n=38)	46–55 yrs (n=8)	χ² (df)	p-value	Cramér's V
YouTube	15 (30.6)	37 (41.6)	13 (34.2)	2 (25.0)	2.27 (3)	0.519	0.11
TikTok	13 (26.5)	22 (24.7)	10 (26.3)	2 (25.0)	0.07 (3)	0.995	0.02
Snapchat	31 (63.3)	56 (62.9)	21 (55.3)	5 (62.5)	0.77 (3)	0.857	0.06
WhatsAppᵃ	3 (6.1)	2 (2.2)	3 (7.9)	2 (25.0)	8.21 (3)	0.042*	0.21
Twitter/Xᵃ	3 (6.1)	4 (4.5)	7 (18.4)	0 (0.0)	8.36 (3)	0.039*	0.21
Instagram	8 (16.3)	25 (28.1)	5 (13.2)	0 (0.0)	6.95 (3)	0.074	0.19

Preference for video-based content was strongly associated with preference for Snapchat, YouTube, TikTok, and Instagram (all p ≤ 0.006; Table 9), but not with WhatsApp or Twitter/X. The association between video preference and Snapchat was particularly strong (χ²(1) = 39.96, p < 0.001, Cramér's V = 0.46), representing a large effect and the strongest effect size observed across all association analyses in this study.

**Table 9:** Association Between Preference for Video-Based Content and Preferred Social Media Platforms (SMPs) (N = 186) * p < 0.05. Note: Cramér's V values were interpreted per Cohen's conventions (small ≈ 0.10, medium ≈ 0.30, large ≈ 0.50). The association between video preference and Snapchat (Cramér's V = 0.46) represents a large effect and is the strongest effect observed across all association analyses in this study.

Preferred SMP	Prefer Video n (%)	Does Not Prefer Video n (%)	χ² (df)	p-value	Cramér's V
YouTube	58 (84.1)	11 (15.9)	17.03 (1)	<0.001*	0.30
TikTok	40 (83.3)	8 (16.7)	10.05 (1)	0.002*	0.23
Snapchat	99 (86.8)	15 (13.2)	39.96 (1)	<0.001*	0.46
WhatsApp	6 (60.0)	4 (40.0)	0.11 (1)	0.740	0.02
Twitter/X	9 (64.3)	5 (35.7)	0.52 (1)	0.470	0.05
Instagram	28 (73.7)	10 (26.3)	7.42 (1)	0.006*	0.20

## Discussion

This study explored patients' preferences for SMPs as channels for receiving educational content during ACLR rehabilitation. The findings confirmed that Snapchat was the most preferred platform, followed by YouTube and TikTok. This preference pattern paralleled the dominant behavioral characteristics of the sample, in which familiarity, daily use, and ease of use were reported as the strongest drivers of platform selection. These findings may inform the selection of communication platforms that align with patient digital habits during ACLR rehabilitation; however, whether platform-aligned education translates into improved learning, adherence, or clinical outcomes cannot be determined from a preference survey alone and requires confirmation through interventional research.

Platforms built around short, visual, and sequential content, often described as micro-video environments, have been proposed to support demonstration-based learning [6]. Micro-video platforms such as Snapchat and TikTok have been identified as accessible channels for health information delivery and engagement in general health contexts [6,7]. It has been suggested that the brief, sequential nature of micro-video content may help focus attention and support engagement during short educational interventions [6], although the applicability of this mechanism to rehabilitation-specific education has not yet been established empirically.

Beyond its use as a passive content channel, Snapchat has been proposed to support asynchronous, informal interactions between users and content creators, which, in rehabilitation contexts, could potentially facilitate brief educational exchanges. However, the present study did not assess two-way communication between patients and clinicians via Snapchat, and no inferences can be drawn regarding interactive follow-up. Given the growing role of telemedicine as a complement to in-person education [22], whether Snapchat can serve as an interactive channel for rehabilitation follow-up remains a hypothesis to be tested in future studies.

Although YouTube has been widely used as a source of health-related information in the United States and Europe [12,16], its educational quality has frequently been rated as suboptimal across multiple surgical and medical domains, and patients' platform preferences are known to vary by region and cultural context. Such preferences are shaped by multiple factors, including platform prevalence within the community, prevailing digital habits, linguistic factors, social communication patterns, and platform-specific privacy characteristics. In the Saudi context, Snapchat is widely used for daily social interaction across diverse population groups, which may help explain its emergence as a preferred channel for accessing health and educational content in the present sample [23,24].

Differences in digital platform usage patterns have been reported in KSA. Snapchat has demonstrated widespread use among large user groups, while YouTube has been described as a secondary preference, consistent with findings that users turn to YouTube for comprehensive and searchable educational resources [25]. TikTok, by contrast, has been characterized as a platform that offers brief, algorithm-driven content [26]. Based on these descriptions and on the present study's finding that YouTube and TikTok were not significantly different in preference after correction for multiple comparisons, we propose, as a framework for future research rather than as a finding derived from this study, that these platforms may occupy complementary rather than competing roles in rehabilitation education: Snapchat for brief, informal, and visually familiar content; YouTube for longer structured instructional material; and TikTok for short motivational or illustrative segments. This framework is hypothesis-generating and requires empirical testing.

The strong preference for Snapchat observed in this study may be consistent with broader literature describing the platform's short-form visual content as supporting timely, relatable communication and user engagement [26,27]. Although the present survey did not assess which specific Snapchat features participants valued, we hypothesize that features such as "stories," which deliver sequential short-form content, could be well-suited to staged rehabilitation content by reducing cognitive load through chunking, and that Snapchat's informal, low-text interface may reduce barriers for patients less engaged with text-heavy platforms [27]. These feature-level hypotheses were not tested in the present study and warrant examination in future work. Additionally, Snapchat's widespread use in Saudi society, including alignment with cultural communication patterns around privacy and direct interpersonal exchange, may further contribute to its preferred status as a channel for health-related content [23,28].

Saudi patients and their families often rely on YouTube and WhatsApp to share health information [10,20]. However, in the current study, patients with ACLR preferred platforms such as Snapchat, TikTok, and YouTube for rehabilitation-related educational content because these platforms provide short, visually clear demonstrations. Similar Saudi studies have indicated the increasing use of these platforms as channels for health awareness among patients and healthcare providers [29].

Unlike some previous studies reporting age-related differences in SMP use, we observed no robust relationship between age and SMP preference [30]. This is most likely explained by the sample's demographic composition: most participants were young adults, and the ≥56 years subgroup (n = 2) was too small to contribute meaningful inferential data and was excluded from subgroup inferential testing. Age-related differences in platform preference, therefore, cannot be ruled out based on this sample; the present study lacked the statistical power and demographic diversity required to detect them reliably. The high Internet penetration in KSA and the active engagement of most users with at least one SMP [8,9] may also have narrowed observable age-related variation in platform choice. Age likely exerts a limited influence in this specific population compared with factors related to content accessibility and platform functional characteristics, but this conclusion should be confirmed in samples with a broader age distribution.

Certain sex-related differences emerged in SMP preferences. Men reported significantly higher preference for Snapchat and TikTok, both of which rely on short-form visual content and rapid communication, whereas women showed a numerically higher preference for Instagram that did not reach statistical significance. These patterns are consistent with reports that fast-paced visual platforms are widely used among young people, particularly for sports and fitness content, whereas Instagram is associated with different patterns of social engagement [10,11,20]. Given the small size of the female subgroup (n = 31), these sex-related findings should be interpreted as preliminary, with confirmation required in more balanced samples.

Across all the demographic subgroups, videos were the preferred format for ACLR rehabilitation education. Audiovisual materials lighten the cognitive load, boost understanding, and improve adherence by facilitating technique demonstrations, which allow patients to observe, imitate, and repeat exercises at their own pace [12,13,16]. In this context, SMPs function as extensions of clinical instruction, supporting the transition from supervised therapy to self-directed rehabilitation. Furthermore, these platforms are free and easily accessible, providing feasible approaches for patients and healthcare providers. Thus, educational and rehabilitation programs can benefit from short, repetitive, and flexible visual content. The observation that video preference was most strongly associated with Snapchat preference, representing the largest effect size identified across all association analyses in this study (Cramér's V = 0.46), suggests that the combination of video-based content and Snapchat delivery, rather than either element alone, may be the clinically most actionable finding of this work. This combined recommendation warrants direct evaluation in future interventional studies.

Source credibility appears to be a fundamental element of engagement, as patients are more willing to follow guidance from authoritative sources such as healthcare professionals [10,20]. Our results support this trend: 79% of participants said that their trust in SMP health content depended on whether it came from a well-known or credible source. Furthermore, people who preferred Snapchat were more likely to trust SMP health information overall and to cite a well-known person as the main reason for their trust. These results suggest that, in the Saudi ACLR context, platform preference is not driven solely by ease of use or content format but also by the perceived authority of the content source.

Taken together, these findings point to a concrete implementation model rather than a generic recommendation. Educational content delivered through patients' preferred platform, in this sample Snapchat, is most likely to achieve meaningful reach when it is (i) created or clinically reviewed by qualified physiotherapists or sports medicine clinicians with expertise in ACLR rehabilitation; (ii) endorsed or disseminated by recognized academic institutions such as university rehabilitation programs or by professional bodies such as the Saudi Physical Therapy Association; and (iii) packaged as short-form video content aligned with the sequential structure of standard ACLR rehabilitation phases. Channeling institutional credibility through a familiar, high-reach platform such as Snapchat, rather than introducing new technologies, may reduce adoption barriers for both patients and clinicians and support scalable, culturally aligned digital education. Whether this model produces measurable improvements in engagement, adherence, or clinical outcomes remains to be tested in controlled trials.

Limitations

This study has several limitations. First, the use of convenience sampling, the self-reported nature of the data, and the cross-sectional design may have introduced recall and social desirability biases, thereby preventing inferences about sustained engagement over time. Second, eligibility was restricted to participants who used more than one SMP and who understood Arabic; these criteria may have introduced selection bias by excluding single-platform users, who could have distinct preferences, and by excluding non-Arabic-speaking residents of Saudi Arabia, which may limit generalizability. Third, because participation occurred through an open Google Form link that was closed when the a priori sample size target was reached, a conventional response rate could not be calculated, and the total number of eligible patients exposed to the invitation is unknown. Fourth, the survey instrument was adapted from prior Saudi studies but was not formally pilot-tested, and no test-retest reliability data are available; while the research team reviewed the adapted items for face validity against the study aims, formal psychometric validation was beyond the scope of this work. Fifth, the sample was predominantly male (83.3%), and the female subgroup (n = 31) was underpowered for reliable inferential analysis, particularly for platforms with low overall preference rates; sex-based findings should therefore be considered preliminary. Potential confounding between sex and age was not addressed through multivariable modeling, given the small female subgroup size and the descriptive aim of the study. Sixth, rehabilitation stage data were not collected, which prevents interpretation of whether platform preferences differ across early, mid, and late ACLR rehabilitation phases. Seventh, the ≥56 years subgroup contained only two participants and was excluded from subgroup inferential testing; this sample therefore lacked the age diversity required to detect potential age-related differences in platform preference, and such differences cannot be ruled out. Finally, although recruitment spanned four Saudi cities (Riyadh, Jeddah, Dammam, and Abha), participants were recruited through 10 private, cash-based physiotherapy and sports rehabilitation clinics, which may have underrepresented patients receiving care through public healthcare pathways or in other regions of the country; findings should therefore be interpreted as applicable to similar private sports rehabilitation populations in major Saudi urban centers, rather than to the KSA population broadly. The study was also conducted within a single cultural context, which may limit direct applicability to regions with different digital and sociocultural environments.

## Conclusions

This study demonstrated that Saudi patients undergoing rehabilitation following ACLR preferred Snapchat as a channel for receiving educational information, followed by YouTube and TikTok. Video-based content was the most preferred educational format, and the credibility of the content source was the most frequently cited determinant of patient trust. The association between video preference and Snapchat preference represented a large effect, the strongest observed in this study, suggesting that the combination of video content delivered through Snapchat may be particularly aligned with patient digital habits in this population. These findings characterize patient preferences and provide a basis for designing platform-aligned educational interventions, but they do not establish that such interventions improve engagement, adherence, or clinical outcomes. Accordingly, the findings are best positioned to inform the design of future controlled studies rather than to directly prescribe clinical practice change. Future research should evaluate whether structured, source-credentialed video content delivered through patients' preferred SMPs can produce measurable benefits in ACLR rehabilitation and should extend this work to more demographically and geographically diverse populations to confirm generalizability.

## Appendices

Supplemental material 1

**Table 10:** Survey Instrument in English

Variable	Question	Response Options
Age	What is your age group?	☐ 18–25 ☐ 26–35 ☐ 36–45 ☐ 46–55 ☐ >55
Gender	What is your gender?	☐ Male ☐ Female
Level of Activity (Pre-Injury)	What was your level of physical activity before the injury?	☐ Inactive (No physical activity) ☐ Intense Activity (Exercise 5 days per week or more) ☐ Light Activity (Daily walking or occasional exercise) ☐ Moderate Activity (Exercise 3 days per week)
Level of Activity (Post-Injury)	What is your current level of physical activity (after injury or surgery)?	☐ Inactive (No physical activity) ☐ Intense Activity (Exercise 5 days per week or more) ☐ Light Activity (Daily walking or occasional exercise) ☐ Moderate Activity (Exercise 3 days per week)
Know/Use SMPs	Which of the following social media platforms do you know or use? (Select all that apply)	☐ YouTube ☐ TikTok ☐ Snapchat ☐ WhatsApp ☐ Twitter/X ☐ Instagram ☐ Facebook ☐ Other: ______
Hours on SMPs (Daily)	How many hours do you spend on social media platforms on average per day?	☐ One hour and less ☐ 2-4 hours ☐ 5-7 hours ☐ 8 hours and more
SMPs (Daily Use)	Which social media platforms do you prefer for daily use? (Select all that apply)	☐ YouTube ☐ TikTok ☐ Snapchat ☐ WhatsApp ☐ Twitter/X ☐ Instagram ☐ Facebook ☐ Other: ______
Hours on SMPs (Health Info / Week)	How many hours per week do you spend searching/read for health-related information on social media?	☐ One hour and less ☐ 2-4 hours ☐ 5-7 hours ☐ 8 hours and more ☐ I do not seek health information through social media platforms
Receive Health Info on SMPs	Which social media platforms do you prefer to receive for health-related content? (Select all that apply)	☐ YouTube ☐ TikTok ☐ Snapchat ☐ WhatsApp ☐ Twitter/X ☐ Instagram ☐ Facebook ☐ I do not search/share ☐ Other: ______
Reasons for Preference	What are the main reasons for your platform preferences? (Select all that apply)	☐ Ease of use ☐ Content variety ☐ Platform popularity ☐ Visual quality ☐ Professional communication ☐ no prefer to use for health: ______
Preferred Content Type	What types of content do you find most effective for receiving rehabilitation education on social media platforms? (Select all that apply)	☐ Videos ☐ Infographics ☐ articles ☐ Live sessions (webinars) ☐ I do not seek to share/receive health information through social media platforms. ☐ Other: ______
Trust in Info	Do you trust the health information provided on social media platforms?	☐ Yes ☐ No ☐ Maybe ☐ Trust depends on the source's credibility
Reason for Trust	Do you trust the information in general, or only if it is from a specialist or doctor you know?	☐ I generally trust. ☐ A well-known person increases my trust in the information ☐ I do not trust social media content
Belief in Educational Use of SMPs	Do you believe in the importance of patient education sessions through social media platforms in any form?	☐ Yes ☐ No ☐ Not sure

Supplemental material 2

**Table 11:** Survey in Arabic The questionnaire was developed in Arabic and administered electronically using Google Forms. All questions were closed-ended with predefined response options, and certain items allowed for multiple selections. For the statistical analysis, social media platform preference variables were coded as binary responses (1 = selected, 0 = not selected). Items that allow multiple selections were treated as non-mutually exclusive variables.

خيارات الإجابة	السؤال	المتغيّر
☐ 18–25 ☐ 26–35 ☐ 36–45 ☐ 46–55 ☐ أكبر من 55	ما هي فئة عمرك؟	العمر
☐ ذكر ☐ أنثى	ما هو جنسك؟	الجنس
☐ غير نشط (بدون نشاط بدني) ☐ نشاط شديد (ممارسة التمارين ≥5 أيام أسبوعيًا) ☐ نشاط خفيف (المشي اليومي أو تمرين متقطع) ☐ نشاط متوسط (ممارسة التمارين 3 أيام أسبوعيًا)	ما هو مستوى نشاطك البدني قبل الإصابة؟	مستوى النشاط قبل الإصابة
☐ غير نشط (بدون نشاط بدني) ☐ نشاط شديد (ممارسة التمارين ≥5 أيام أسبوعيًا) ☐ نشاط خفيف (المشي اليومي أو تمرين متقطع) ☐ نشاط متوسط (ممارسة التمارين 3 أيام أسبوعيًا)	ما هو مستوى نشاطك البدني الحالي (بعد الإصابة أو الجراحة)؟	مستوى النشاط بعد الإصابة
☐ يوتيوب ☐ تيك توك ☐ سناب شات ☐ واتساب ☐ إكس تويتر ☐ إنستغرام ☐ فيسبوك ☐ أخرى	أي من منصات التواصل الاجتماعي التالية تعرفها أو تستخدمها؟ (اختر كل ما ينطبق)	معرفة/استخدام المنصات
☐ ساعة أو أقل 2–4 ☐ 5–7☐ 8 ☐ ساعات أو أكثر	كم ساعة تقضي يوميًا في استخدام منصات التواصل الاجتماعي؟	الوقت اليومي على المنصات
☐ يوتيوب ☐ تيك توك ☐ سناب شات ☐ واتساب ☐ إكس (تويتر) ☐ إنستغرام ☐ فيسبوك ☐ أخرى	أي من منصات التواصل الاجتماعي التالية تفضل استخدامها يوميًا؟ (اختر كل ما ينطبق (	المنصات المفضلة للاستخدام اليومي
☐ ساعة أو أقل ☐ 2–4☐ 5–7☐ 8ساعات أو أكثر ☐ لا استخدم منصات التواصل الاجتماعي للبحث عن المعلومات الصحية	كم ساعة أسبوعيًا تقضي في البحث/قراءة المعلومات الصحية عبر منصات التواصل الاجتماعي؟	الوقت الأسبوعي للبحث عن المعلومات الصحية
☐ يوتيوب ☐ تيك توك ☐ سناب شات ☐ واتساب ☐ إكس (تويتر) ☐ إنستغرام ☐ فيسبوك ☐ لا أبحث/أشارك ☐ أخرى: ______	أي من المنصات التالية تفضل للحصول على المعلومات الصحية؟ (اختر كل ما ينطبق (	المنصات المفضلة للحصول على المعلومات الصحية
☐ سهولة الاستخدام ☐ تنوع المحتوى ☐ شعبية المنصة ☐ جودة المحتوى البصري ☐ التواصل المهني ☐ لا يفضل استخدامها للمعلومات الصحية	ما هي الأسباب الرئيسية لتفضيلك لمنصة معينة؟ (اختر كل ما ينطبق (	أسباب التفضيل
☐ فيديوهات ☐ رسوم بيانية ☐ مقالات ☐ جلسات مباشرة ندوات عبر الإنترنت ☐ لا أستخدم وسائل التواصل للمشاركة ☐ أخرى: ______	ما نوع المحتوى الذي تراه الأكثر فاعلية لتلقي التعليم حول إعادة التأهيل عبر وسائل التواصل الاجتماعي؟ (اختر كل ما ينطبق (	نوع المحتوى المفضل
☐ نعم ☐ لا ☐ ربما ☐ تعتمد الثقة على مصداقية المصدر	هل تثق بالمعلومات الصحية المتوفرة عبر منصات التواصل الاجتماعي؟	الثقة بالمعلومات
☐ أثق بشكل عام ☐ الثقة تزداد إذا كان المصدر معروفًا أو مختصًا ☐ لا أثق بمحتوى وسائل التواصل الاجتماعي	هل تثق بالمعلومات بشكل عام، أم فقط إذا كانت من مختص أو طبيب تعرفه؟	سبب الثقة
☐ نعم ☐ لا ☐ غير متأكد	هل تؤمن بأهمية تقديم جلسات تعليمية للمرضى عبر منصات التواصل الاجتماعي بأي شكل؟	الإيمان بالتعليم عبر المنصات
